# A scalable, high-throughput glue perturbation screening platform for molecular glue discovery

**DOI:** 10.1016/j.isci.2026.117600

**Published:** 2026-09-19

**Authors:** Diyun Huang, Zeyu Shuang, Lu Chen, Hanyue Ouyang, Peiyu Yang, Lichang Huang, Wenchao Lu, Ming Shi, Xiaofan Ding, Baishan Jiang, Wenchao Wu

**Affiliations:** 1 State Key Laboratory of Oncology in South China, Guangdong Provincial Clinical Research Center for Cancer, Guangdong Key Laboratory of Nasopharyngeal Carcinoma Diagnosis and Therapy, Sun Yat-sen University Cancer Center, Guangzhou 510060, P.R. China; 2 Department of Breast Oncology, State Key Laboratory of Oncology in South China, Guangdong Provincial Clinical Research Center for Cancer, Sun Yat-sen University Cancer Center, Guangzhou 510060, P.R. China; 3 Department of Radiation and Medical Oncology, Medical Research Institute, Frontier Science Center of Immunology and Metabolism, Zhongnan Hospital of Wuhan University, Wuhan University, Wuhan 430071, China; 4 Department of Hepatobiliary Oncology, State Key Laboratory of Oncology in South China, Guangdong Provincial Clinical Research Center for Cancer, Sun Yat-sen University Cancer Center, Guangzhou 510060, P.R. China; 5 Lingang Laboratory, Shanghai 200031, China; 6 MOE Frontiers Science Center for Precision Oncology, University of Macau, Macau SAR, China

**Keywords:** molecular glue degraders, targeted protein degradation, TPD, high-throughput screening platform, CRBN, E3 ligase, drug discovery

## Abstract

The discovery of molecular glue degraders (MGDs) is undergoing a paradigm shift from serendipitous discovery toward empirical design. However, efficiently prioritizing active leads from synthetic libraries while simultaneously confirming their E3 dependency remains a formidable challenge. In this study, we introduce the glue perturbation screening (GPS) platform, a conceptual and technical advance that integrates high-throughput, low-cost Digital RNA with pertUrbation of Genes 2 (DRUG-seq2) with isogenic cereblon (CRBN) wild-type/knockout (WT/KO) models. The significance of our work lies in the development of a multi-dimensional readout that digitizes compound activity and mechanism in a single step. By mapping transcriptomic perturbations onto a coordinate system of differential expression and gene set enrichment, we establish a tiered prioritization strategy: distinguishing high-potency glues from non-specific toxic molecules and recognizing latent biological activities in sub-threshold compounds that guide further structural optimization. This platform provides a scalable, mechanism-aware roadmap for the rational discovery of next-generation degraders, offering a powerful tool to accelerate the targeting of “undruggable” proteins.

## Highlights

• GPS integrates DRUG-seq2 with CRBN WT/KO models for molecular glue screening
• Transcriptomic profiling enables tiered prioritization of molecular glue candidates
• LC-02-047-P1 is identified as a novel CRBN-dependent CK1α-degrading molecular glue

## Graphical abstract

**Figure:**
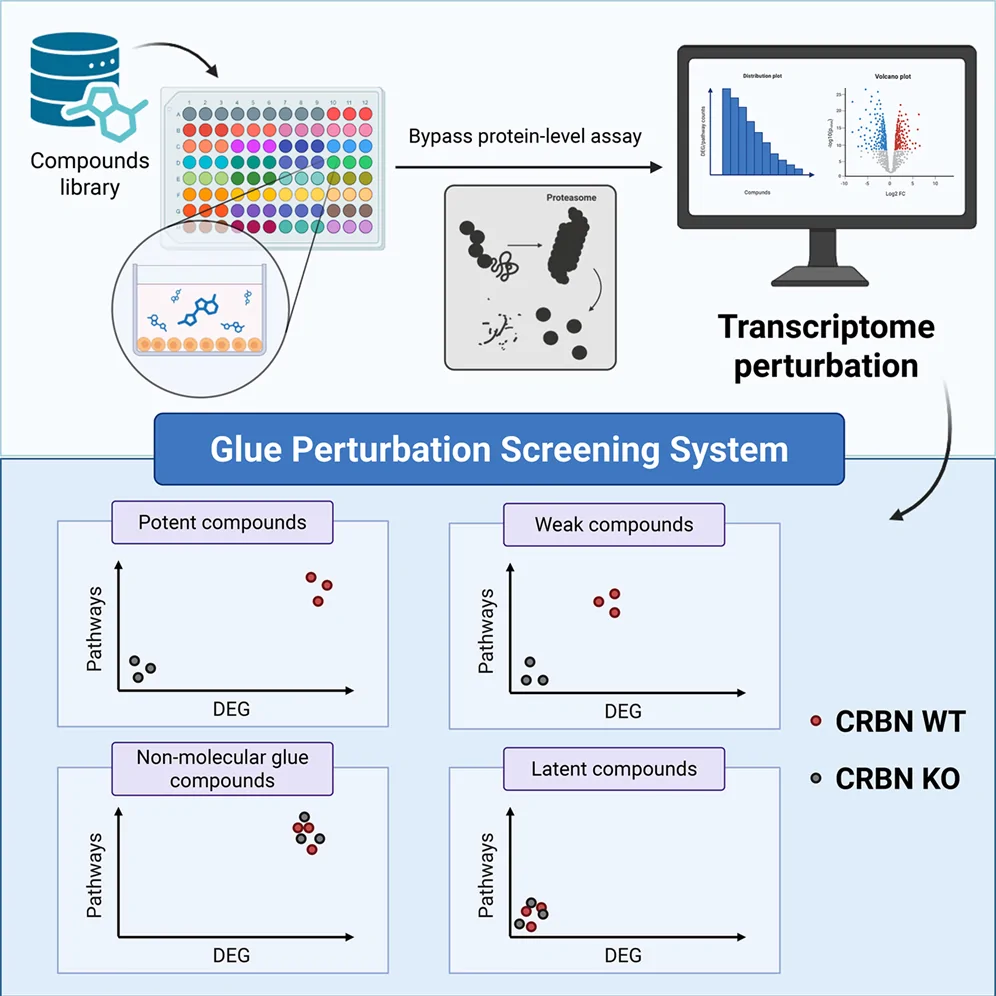

## Teaser

Biological sciences; Biochemistry; Omics; Biological sciences research methodologies

## Introduction

The field of targeted protein degradation (TPD) has fundamentally shifted the paradigm of drug discovery from “occupancy-driven” inhibition to “event-driven” protein depletion.^1^ Among the various TPD modalities, molecular glue degraders (MGDs) have emerged as a particularly potent strategy. Unlike proteolysis-targeting chimeras (PROTACs), which are large, bivalent molecules, MGDs are typically small, monovalent compounds, offering superior pharmacokinetic properties and membrane permeability.^2^ Most importantly, MGDs exert their function by remodeling the surface of an E3 ubiquitin ligase to create a neo-interface, enabling the recruitment and subsequent degradation of proteins that lack traditional druggable pockets, such as transcription factors and scaffold proteins.^3^

Historically, the discovery of MGDs has been largely serendipitous. Iconic examples, such as the clinical success of immunomodulatory drugs (IMiDs)^4^ and the sulfonamide indisulam,^5^ were only characterized as molecular glues years after their initial phenotypic observations. However, relying on serendipity is insufficient for the systematic expansion of the degradable proteome. To explore the full therapeutic potential of MGDs, there is an urgent need to transition from retrospective mechanistic studies to prospective, high-throughput screening of diverse synthetic chemical libraries.

Current screening strategies for MGDs, while effective in specific contexts, face significant practical bottlenecks that hinder their application in early-stage discovery. Proteomics-based approaches, for instance, provide a comprehensive view of perturbation of protein abundance but remain cost-prohibitive and limited by low throughput, making them impractical for the primary screening of large-scale chemical libraries.^6^^,^^7^ Similarly, target-based biochemical assays, such as AlphaLISA or time-resolved fluorescence resonance energy transfer (TR-FRET), necessitate purified proteins and *a priori* knowledge of the target-E3 interaction, which contradicts the objective of identifying glues for previously “undruggable” or unknown neosubstrates.^8^ Furthermore, while phenotypic screening remains biologically relevant, it often fails to distinguish true degraders from traditional inhibitors, requiring laborious and time-consuming downstream deconvolution of the mechanism of action (MoA).^9^^,^^10^ Consequently, there is a critical need for a cost-effective and high-resolution primary screening method that can capture the functional signature of a degrader to accelerate the MGD discovery process.

Transcriptomic profiling has recently emerged as a powerful “universal readout” for drug activity. A compelling proof-of-concept is seen in the study of IMiD resistance: while earlier-generation glues like lenalidomide and pomalidomide fail to elicit biological responses in resistant cell lines, the next-generation degrader CC-92480 (mezigdomide) induces profound transcriptional remodeling and IKZF1 degradation.^11^ This approach is supported by the growing success of transcriptomics-based drug discovery.^12^ Technologies such as L1000 have demonstrated that gene expression signatures can serve as high-dimensional proxies for cellular states.^13^ More recently, the development of DRUG-seq has significantly reduced the cost and labor associated with RNA sequencing (RNA-seq) by utilizing direct cell lysis and multiplexed library preparation.^14^^,^^15^ These advancements provide a solid foundation for using transcriptomic “fingerprints” to bypass the limitations of traditional protein-level assays.

In this study, we present a transcriptomic-driven workflow for the identification and mechanistic characterization of cereblon (CRBN)-dependent molecular glues. Utilizing DRUG-seq2, a miniaturized RNA-seq platform, we profiled the transcriptional responses of 117 synthetic molecules across isogenic cell lines to directly visualize the E3-mediated transcriptional shifts and successfully identified LC-02-047-P1 as a lead compound. This framework was designed not only to quantify the potency of candidate compounds but also to map their downstream biological consequences through comprehensive gene-set enrichment. By defining a tiered classification system based on genetic dependency and functional coordination, we demonstrate how transcriptomic profiling can be utilized to uncover latent bioactivity and prioritize “seeds” for further chemical optimization.

## Results

### Profiling CRBN-modulating libraries via newly defined GPS system

To systematically evaluate the bioactivity and mechanisms of molecular glue libraries, we implemented an integrated, high-throughput transcriptomic screening workflow (Figures 1A and 1B). Our diverse library comprises 117 compounds rationally designed to modulate CRBN E3 ligase activity, encompassing 11 commercial molecular glues and 106 in-house candidate compounds synthesized by two collaborative research groups. All 117 compounds were evaluated at a uniform concentration (1 μM) for 24 h in 96-well plates, with each tested in biological triplicate (Figure 1A).

**Figure 1:**
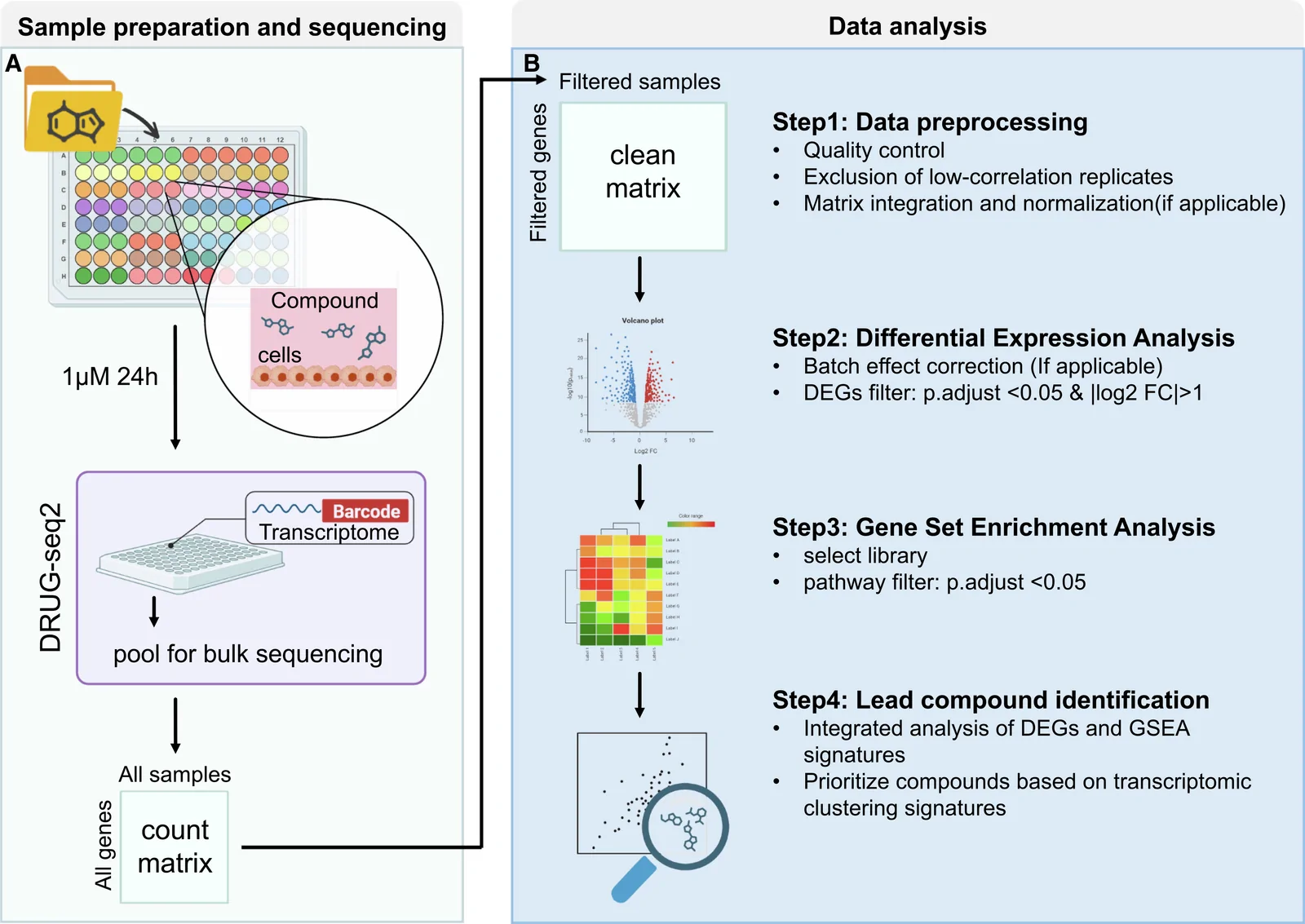
Profiling CRBN-modulating libraries via an optimized high-throughput transcriptomic screening system (A) Schematic representation of the experimental design. Hep3B WT and *CRBN*-KO cells were seeded in 96-well plates and treated with a library of 117 compounds at a uniform concentration of 1 μM for 24 h. DMSO were utilized as negative control with 9 biological replicates per plate. Following treatment, cells were processed via the DRUG-seq2 sequencing protocol, featuring direct lysis and high-throughput library preparation without RNA purification. (B) Overview of the bioinformatic analysis pipeline. Raw sequencing data were subjected to preprocessing and quality control to generate a gene expression matrix. Downstream analyses included differentially expressed gene (DEG) analysis using DESeq2 (criteria: |log2FC| > 1 and padj < 0.05) and gene set enrichment analysis (GSEA). Finally, candidate compounds were prioritized based on their ability to induce specific transcriptomic signatures. See also Figure S1.

To achieve cost-effective and accessible profiling, we leveraged the optimized DRUG-seq2 platform.^16^ Building upon the foundational principles of forgoing RNA purification and employing a multiplexing strategy,^14^ DRUG-seq2 significantly enhances throughput while dramatically reducing per-sample costs. Moreover, by incorporating refined library construction modules, this platform offers superior sensitivity in capturing low-abundance regulatory transcripts, a feature crucial for capturing the subtle perturbation induced by immature molecular glues.^16^

Prior to formal downstream analysis, we implemented a rigorous intra-plate quality control step for the DMSO replicates to ensure a high-fidelity transcriptional baseline for the identification of pharmacological signals. Given the direct-lysis protocol of DRUG-seq2, establishing a stable reference state is critical to minimize technical noise and maximize the sensitivity for detecting low-magnitude differential gene expression. For each 96-well plate, we performed a systematic pairwise Pearson correlation analysis across all DMSO wells to evaluate their technical consistency (Figure S1). Replicates exhibiting a correlation coefficient (r < 0.95) were identified as technical outliers and were subsequently excluded from the baseline pool. By constructing the reference transcriptome solely from high-consensus DMSO wells, we established a robust and self-consistent “zero-point,” providing the necessary statistical power to resolve subtle transcriptomic perturbations induced by the molecular glue library.

The subsequent bioinformatic analysis was designed to facilitate the primary triage of active molecules (Figure 1B). Rather than relying on a single readout, we employed a multi-dimensional approach integrating differential expression analysis (DEA) and gene set enrichment analysis (GSEA). This framework allowed us to stratify compounds based on their transcriptional vigor and, more importantly, to preliminarily assess their mechanistic dependency on the target E3 ligase. By comparing the transcriptional signatures of unknown compounds against the benchmark and subsequent genetic perturbation models, we could efficiently distinguish active degraders and prioritize those exhibiting specific E3-dependent response patterns.

### Construction and functional validation of a CRBN-deficient genetic model

To establish a robust system for identifying CRBN-dependent molecular glues, we generated an isogenic cell pair to serve as a genetic benchmark. We utilized liver cancer cells Hep3B to construct a stable cell line expressing Cas9, which would serve as the *CRBN* wild-type control (hereafter referred to as “WT”) that provided a consistent genetic background for subsequent CRISPR-mediated editing (Figure 2A). To generate a corresponding *CRBN* knockout (KO) model, we introduced single-guide RNA (sgRNA) targeting the *CRBN* gene into the WT background, followed by single-cell isolation and clonal expansion (Figure 2A). Among the four candidate clones (clone1–clone4) evaluated, clone3 and clone4 demonstrated the most consistent depletion of the CRBN protein while maintaining stable Cas9 expression (Figures 2B–2D).

**Figure 2:**
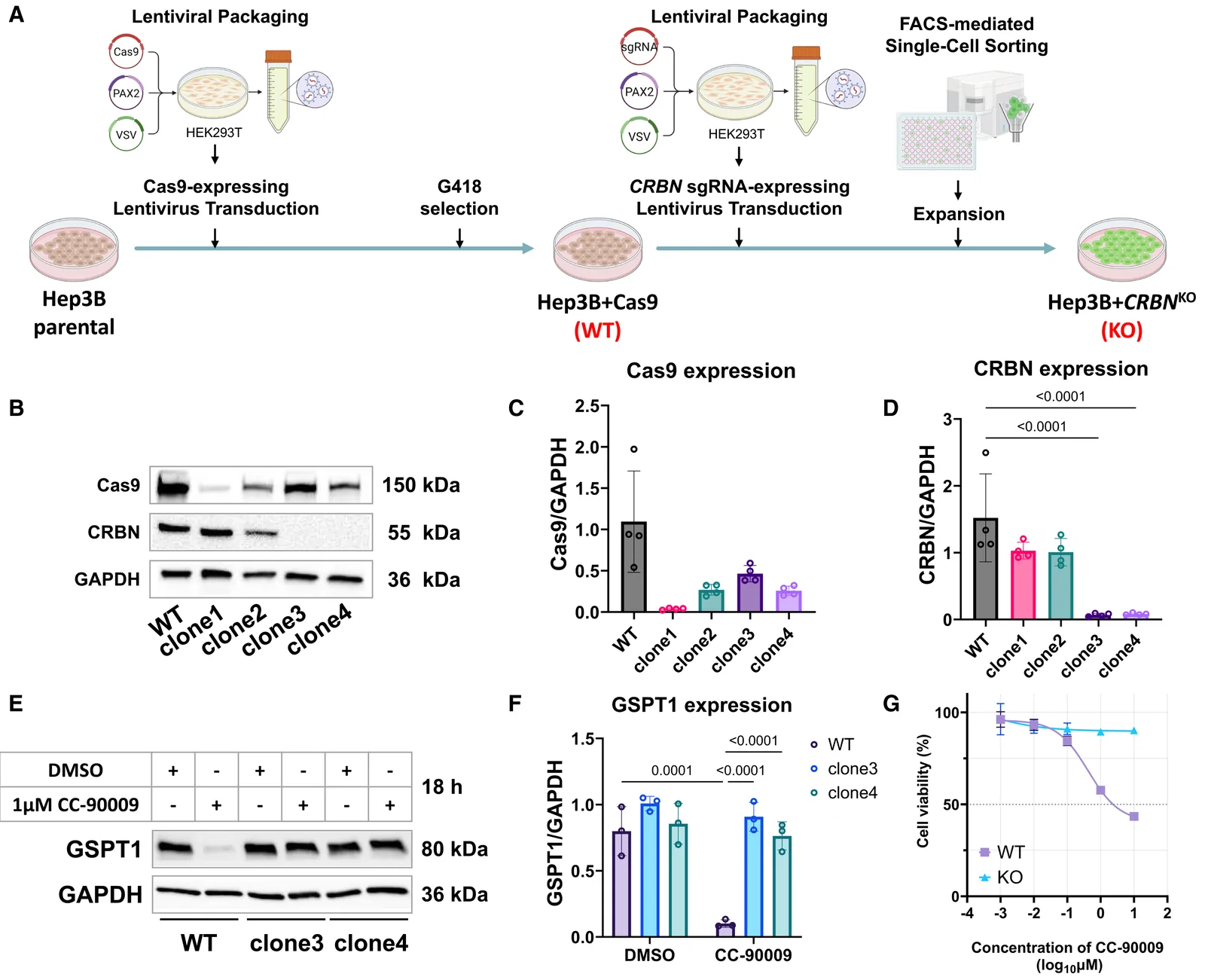
Construction and functional validation of an isogenic CRBN-knockout cell model (A) Schematic of the CRISPR-Cas9-mediated gene editing strategy. The Hep3B cell line stably expressing Cas9 (defined as WT) was transfected with sgRNAs targeting *CRBN*, followed by single-cell isolation and clonal expansion. (B–D) Screening of single-cell clones via western blot. Expression levels of CRBN and Cas9 were assessed in four selected clones (clone1–clone4). Data were from four independent biological experiments. Significant *p* values are indicated above the brackets, evaluated by two-way ANOVA. (E and F) Blockade of the molecular glue action pathway in CRBN-deficient cells. WT, clone3, and clone4 cells were treated with CC-90009 at the indicated concentration and time point. GSPT1 protein levels were analyzed by western blot. Data were from three independent biological experiments. Significant *p* values are indicated above the brackets, evaluated by two-way ANOVA. (G) Pharmacological validation via cell viability assays. Dose-response curves of WT and *CRBN*-KO (clone3) cells treated with CC-90009 for 72 h. Data are presented as mean ± SEM from *n* = 4 biological replicates.

To further verify the functional loss of the CRBN-mediated degradation pathway, we performed a pharmacological challenge using CC-90009, one of the aforementioned commercial molecular glues. CC-90009 is a potent GSPT1-degrading molecular glue whose cytotoxic effect is strictly contingent upon its recruitment to the CRBN E3 ligase complex.^17^ We first examined the degradation of the substrate at the protein level. CC-90009 can rapidly and almost completely degrade GSPT1 in WT cells, and this degradation effect is completely eliminated by the KO of *CRBN*. (Figures 2E and 2F). Consistent with these readouts, cell viability assays further confirmed the functional deficiency of the CRBN complex. While WT cells remained highly sensitive to CC-90009-induced apoptosis, the clone3 exhibited profound resistance characterized by a significant right-shift in the half maximal inhibitory concentration (IC_50_) curve and a failure to trigger growth inhibition (Figure 2G). Based on the robust loss of both substrate degradation and pharmacological response, clone3 was selected as the definitive KO cell line for all subsequent transcriptomic-based E3-dependency screening.

The establishment of this *CRBN* WT/KO pair provides a definitive platform for our transcriptomic screening. It allows us to distinguish whether the transcriptional perturbations induced by in-house compounds are true E3-dependent events, which should be abolished in the KO cells, or if they stem from off-target effects or other non-degradation-related mechanisms.

### Transcriptomic profiling effectively identifies molecular glue bioactivity and mechanistic signatures

To evaluate the bioactivity and distinctive response patterns of the 117 compounds library, we first performed DRUG-seq2-based profiling in WT cells. As shown in Figure 3A, the library exhibited a highly polarized activity spectrum. About 20% of the library remained transcriptionally inert, exhibiting zero differentially expressed genes (DEGs) at the tested concentration (1 μM, 24 h). In contrast, the active compounds were clearly stratified based on their response intensity. Notably, the high-activity cluster (DEG >50) was dominated by well-known MGDs, including SJ3149 (target CK1α),^18^ lenalidomide (target CK1α and IKZF1),^19^^,^^20^ BMS-986397 (target CK1α),^21^ and MYC degrader 1 (target GSPT1 and MYC),^22^ with SJ3149 eliciting the most profound transcriptional response (Figure 3A).

**Figure 3:**
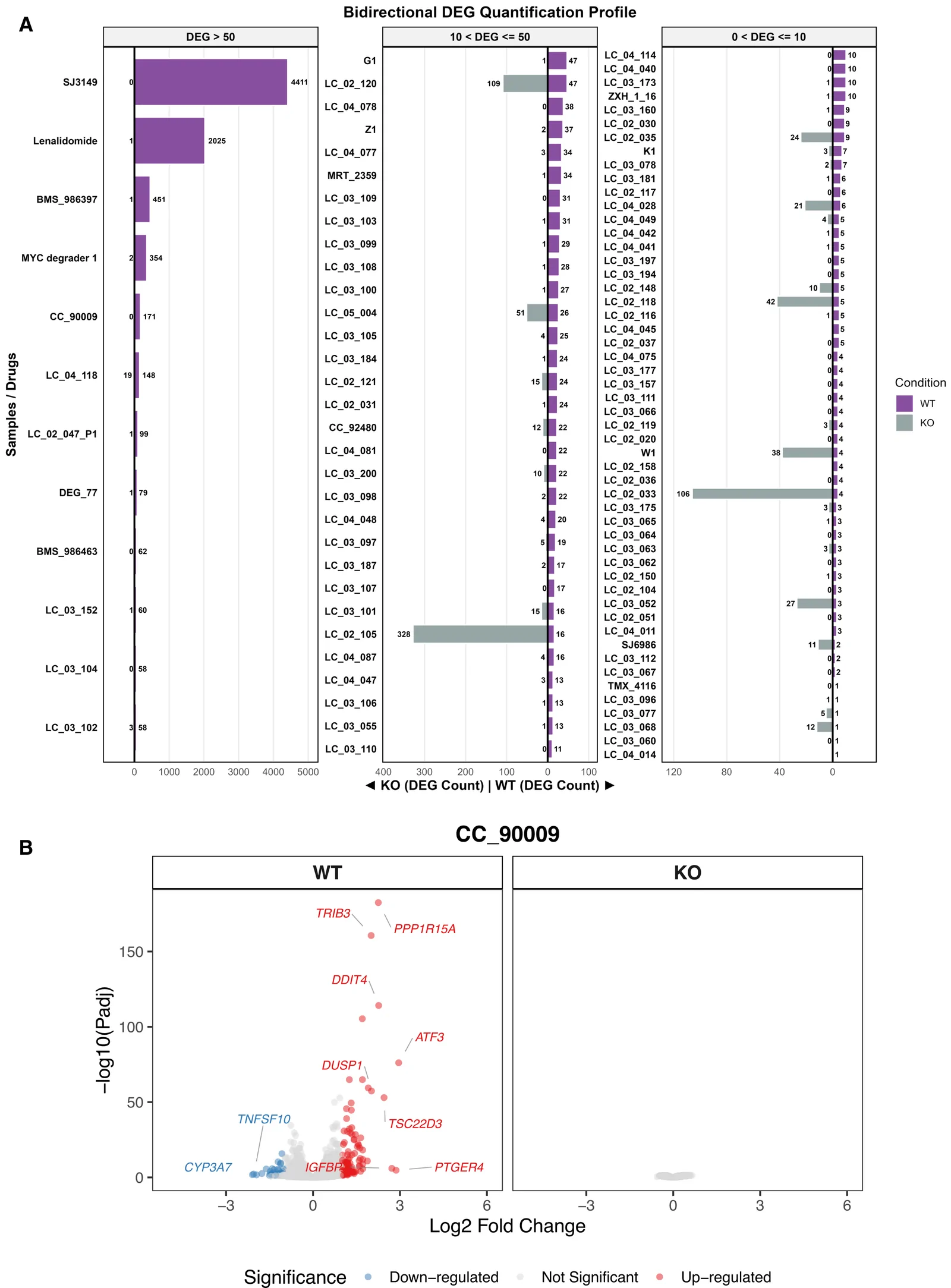
Systematic transcriptomic profiling defines compound potency and CRBN dependency (A) Quantitative distribution of transcriptional responses across the compound library. The bidirectional bar chart displays the number of differentially expressed genes (DEGs) induced by each compound at 1 μM for 24 h in wild-type (WT, purple bars) and CRBN-knockout (KO, gray bars) Hep3B cells. Compounds are categorized into three sections based on their DEG counts in WT cells (DEG >50, 10 < DEG <50, and 0 < DEG <10). Compounds with zero DEGs in both conditions are omitted from the visualization. (B) Volcano plots of CC-90009 in WT (left) and *CRBN*-KO (right) cells. The top 10 significantly regulated genes are annotated with their gene symbols. All compound-treatment transcriptomic data were generated from three independent biological replicates per condition (*n* = 3).

Intriguingly, the comparative analysis between WT and KO cells provides a powerful filter to distinguish genuine CRBN-dependent molecular glues from other bioactive molecules. For the majority of high-activity compounds (e.g., SJ3149 and lenalidomide), their transcriptional activity was almost completely abolished in *CRBN*-KO cells, robustly demonstrating a strict reliance on the CRBN E3 ligase machinery—a hallmark of classic molecular glues. We were also surprised to see that one of the in-house synthesized compounds, LC-02-047-P1, was among the potent molecular glues with high activity, implying that it is highly likely to be a promising new molecular glue compound. Conversely, a subset of compounds exhibited a CRBN-independent profile. For instance, LC-02-105 and LC-02-033 displayed a paradoxical “KO-amplified” phenotype. Such CRBN-resistant or enhanced transcriptional responses strongly indicate that these compounds function via CRBN-independent mechanisms. They may represent non-degrader bioactive molecules, such as direct kinase inhibitors or cytotoxic agents, or degraders that hijack alternative E3 ligases (e.g., VHL or DCAF16) rather than CRBN. Regardless of the potential non-specific mechanisms, this has enabled us to rule out compounds whose systemic toxicity or intrinsic effects of the scaffold were stronger than the degradation function. Taken together, these distinct response patterns underscore that our profiling system serves as a highly discriminative platform capable of effectively segregating bona fide CRBN-dependent molecular glues from the background of non-specific or alternative-mechanism compounds.

We further investigated the transcriptional characteristics of potent active candidates to assess whether our analysis process could capture the mechanistically relevant signals. We observed a series of regulatory targets of ATF4 among the top 10 genes that were upregulated, including the master integrated stress response (ISR) regulators *DDIT3* (CHOP), the stress-responsive cytokine GDF15, and factors involved in feedback inhibition and growth control such as *TRIB3*, *DDIT4* (REDD1), and *PPP1R15A* (GADD34) (Figure 3B).^23^^,^^24^ Upon CC-90009-mediated GSPT1 depletion, the resulting impairment of translation termination leads to activation of the ISR pathway and the subsequent preferential translation of the key transcription factor ATF4.^25^ This is highly consistent with our Gene Ontology (GO) enrichment analysis results, where significant enrichment was observed for the ISR, endoplasmic reticulum stress (ERS), unfolded protein response (UPR), apoptosis, and other related pathways (Figure 4A). We conducted a similar analysis on the candidate compound LC-02-047-P1. In contrast, LC-02-047-P1 exhibited regulatory characteristics related to metabolism, rather than the stress characteristics of CC-90009 (Figure 4B). This may suggest that its target for degradation could be an upstream switch in multiple metabolic pathways within the cell.

**Figure 4:**
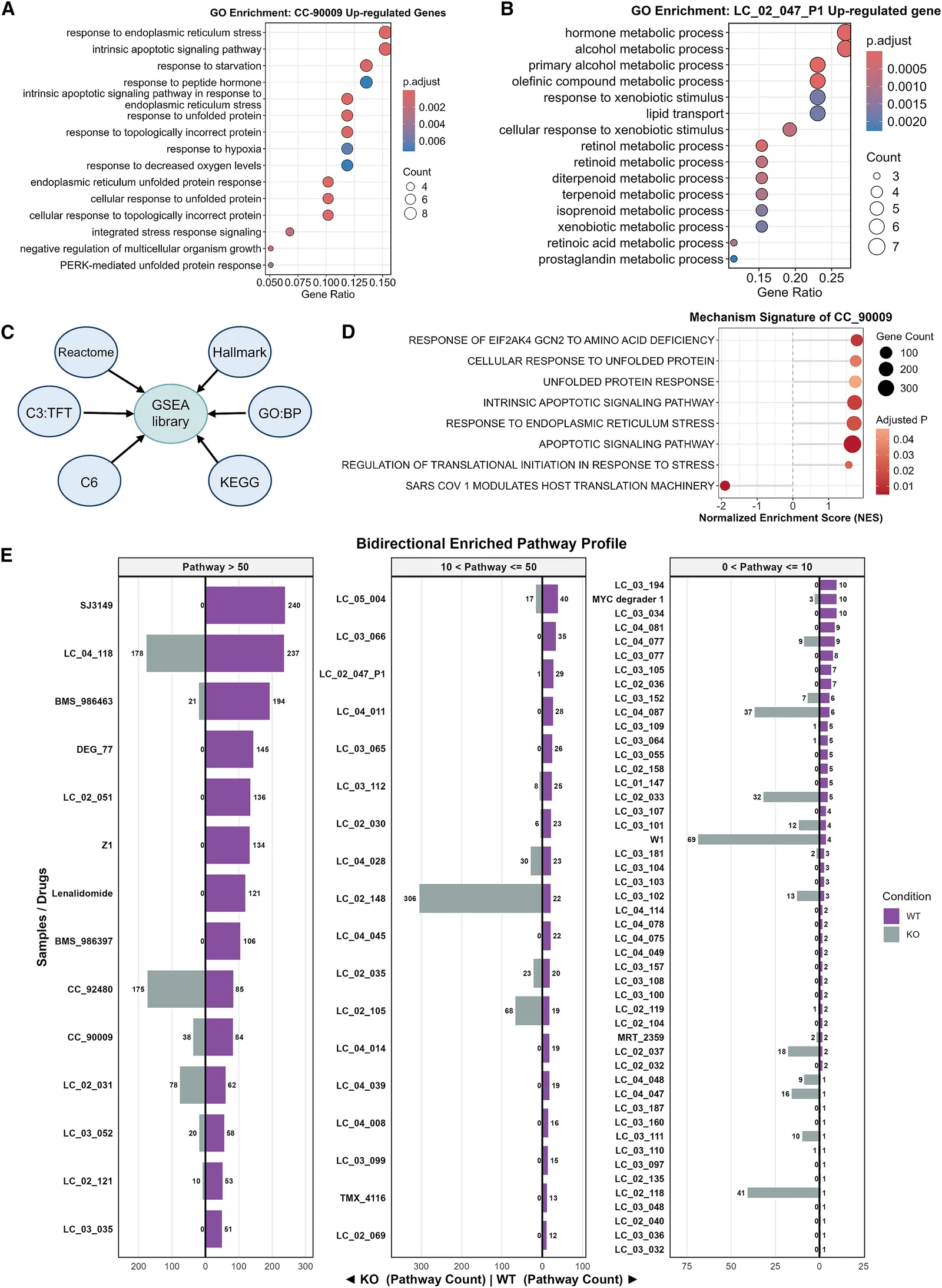
Systemic functional enrichment analysis reveals latent bioactivity and mechanistic diversity (A and B) Distinct biological response patterns of potent candidates. Gene Ontology (GO) enrichment analysis of significantly upregulated genes induced by (A) CC-90009 and (B) LC-02-047-P1. (C) Schematic of the customized GSEA library construction. (D) Deep-dive GSEA profiling of the benchmark glue CC-90009. (E) Quantitative distribution of GSEA across the compound library. The bidirectional bar chart displays the number of pathways in WT (purple bars) and KO (gray bars) Hep3B cells. Compounds are categorized into three sections based on their pathway counts in WT cells (pathway >50, 10 < pathway <50, and 0 < pathway <10). Compounds with zero pathways in both conditions are omitted from the visualization. All compound-treatment transcriptomic data were generated from three independent biological replicates per condition (*n* = 3).

In summary, this transcriptomic framework provides a powerful tool to not only triage molecules based on their primary E3 dependency but also to uncover unique biological signatures that differentiate candidates with diverse modes of action.

### Functional enrichment and GSEA profiling further uncover latent molecular glue activities

Recognizing that many compounds in our library exhibited minimal or zero DEGs at the standard threshold, we reasoned that these “transcriptionally silent” molecules might still harbor sub-threshold biological activity that could be captured through coordinated gene-set shifts. To maximize our ability to capture sub-threshold biological signals, we established a customized GSEA library by integrating six authoritative gene-set collections, including Hallmark, Reactome, Kyoto Encyclopedia of Genes and Genomes (KEGG), Gene Ontology Biological Process (GO:BP), C6, and C3 collection: Transcription Factor Targets (C3:TFT) (Figure 4C). This consolidated resource allows us to quantify the subtle but statistically significant functional perturbation breadth of each compound that might otherwise be missed by discrete gene-level analysis.

A library-wide GSEA assessment in WT cells revealed that several compounds that yielded zero DEGs in the primary analysis showed significant enrichment in multiple GSEA pathways (Figure 4E). This “hidden signal” suggests that these molecules possess a nascent biological activity that, while currently weak, provides a valuable starting point for further medicinal chemistry optimization to enhance their potency and specificity. To validate the biological relevance of these signals, we scrutinized the GSEA profile of CC-90009 as a deep-dive exemplar (Figure 4D). The results also displayed a robust upregulation of ISR, proteasome, and apoptotic pathways. Notably, we observed a profound downregulation of translation-related machineries. This corresponds precisely to the biological role of GSPT1 as a translation termination factor. Its degradation leads to ribosome stalling at stop codons, generating ribosome collisions that are sensed by stress response pathways such as the ZAKα-mediated ribotoxic stress response.^26^ This triggers compensatory transcriptional feedback, such as the ISR, to suppress the cellular translation apparatus and mitigate proteotoxic stress.^27^

Similarly, we evaluated the CRBN dependency of these functional signatures by comparing the number of significant GSEA pathways in WT and KO backgrounds (Figure 4E). For traditional IMiDs and molecular glues such as lenalidomide, the transcriptional response was completely abolished in the KO group, presenting us with the distinct characteristic manifestations of the real molecular glue in our system. We also noticed the candidate molecule LC-02-051. Although it did not perform particularly well in the DEG analysis, it ranked among the top in the pathway enrichment analysis. Moreover, CRBN KO completely blocked the transcriptional changes it caused, suggesting that LC-02-051 is likely a CRBN-dependent molecular glue and has the advantage of further optimization.

However, for CC-92480, we observed a slight increase in the number of enriched pathways in the KO group. We speculate that this might be due to the fact that the degradation target does not exist in the Hep3B cell line. In WT cells, the lack of the high-affinity target (IKZF1) likely drives the CC-92480/CRBN complex to bind secondary, non-canonical substrates, eliciting an on-mechanism yet off-target transcriptional change. Conversely, in *CRBN*-KO cells, where both the target and the requisite E3 ligase component are absent, the uncomplexed compound may act as a redundant chemical stressor. This may shift the cellular response toward off-mechanism xenobiotic toxicity, triggering widespread, non-specific stress and compensatory pathways that paradoxically expand the numbers of enriched terms. This highlights the importance of cell-background context when evaluating the specificity of molecular glue candidates.

In conclusion, enrichment analysis provides a further basis for the stratification of compound screening. It is worth noting that DEA is worthy of being prioritized as the main reading for candidate identification, as it offers superior interpretability and direct quantitative measurement of the direct biological effects of the compounds. And GSEA, as its downstream process, further captures the potential activity in sub-threshold molecules and effectively eliminates completely inert compounds.

### Multi-dimensional stratification and hit prioritization of the molecular glue library

To synthesize the complex transcriptomic and functional data into a unified evaluative framework, we constructed a multi-dimensional scatterplot integrating DEG counts and pathway enrichment breadth (Figure 5A). The resulting distribution reveals a distinct pharmacological gradient across the library. We conclude that in this coordinate system, the positions of compounds and their migration behavior upon CRBN knockdown directly reflect their pharmacological potency and MoA. Strong molecular glues naturally favor the right and upper right quadrants of the graph, representing a powerful driving ability to simultaneously alter a large number of DEGs and functional pathways. Both the mature molecular glues, such as SJ3149 and lenalidomide, as well as the LC-02-047-P1 and CC-90009 of our interest, fit this distribution characteristic. In contrast, compounds with moderate or weak activity were clustered in the upper left quadrant. These molecules are characterized by low absolute DEG counts, but they have a broad impact on biological pathways. Notably, these active signals were completely abolished after CRBN knockdown, resulting in a sudden migration of their data points to the origin in the bottom left corner, reflecting their strict dependence on the CRBN mechanism for phenotypic and transcriptomic efficacy, fulfilling the classical criteria for targeted driving molecular collagen degraders.

**Figure 5:**
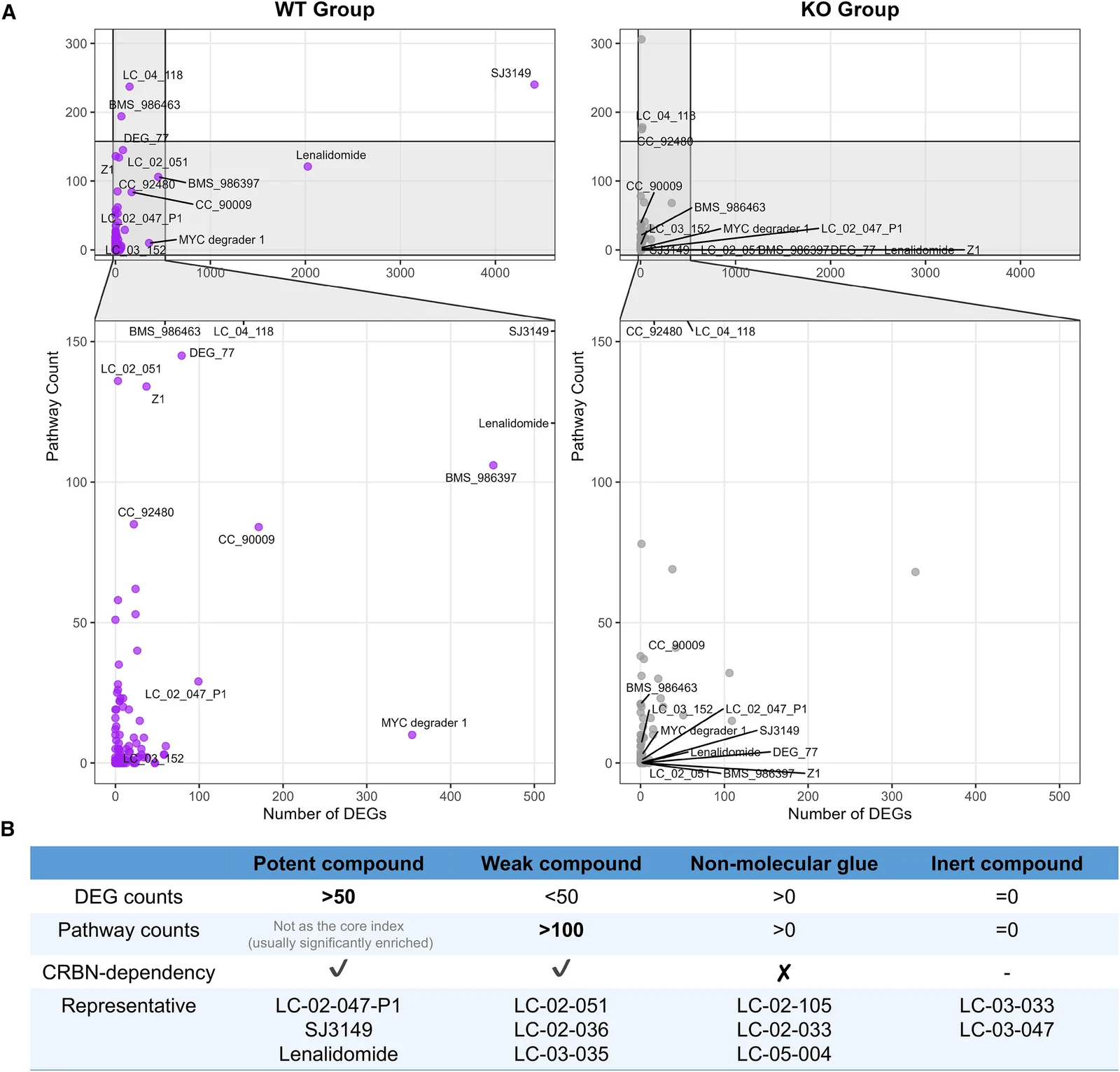
Multi-dimensional integration and tiered stratification of molecular glue candidates (A) Multi-dimensional landscape of pharmacological perturbations. Scatterplot integrating transcriptomic potency (number of DEGs, *x* axis) and functional breadth (number of enriched GSEA pathways, *y* axis) across the compound library in WT and *CRBN*-KO Hep3B cells. (B) Tiered classification system for lead prioritization. Compounds are stratified into four distinct archetypes based on their integrated performance across the screening pipeline. All compound-treatment transcriptomic data were generated from three independent biological replicates per condition (*n* = 3). See also Table S1.

Beyond identifying active degraders, this spatial framework serves as a filter to segregate “non-glue” and inactive library members. Compounds that consistently aggregate near the origin across both the WT and KO group represent pharmacologically inert molecules that fail to elicit any meaningful transcriptomic response. In sharp contrast, a minor subset of compounds remains far removed from the origin, maintaining their elevated DEG counts or pathway breadths in both experimental groups regardless of the CRBN status. The persistent, non-shifting distribution of these outliers flags them as target-independent agents or conventional inhibitors whose cytotoxic and phenotypic driving forces bypass the specialized degradation pathway entirely, thereby providing a clear visual segregation of true molecular glue activity from generalized off-target effects.

Based on this integrated workflow, we established a tiered classification system to prioritize lead candidates for subsequent development (Figure 5B). We utilized 3 key readouts—transcriptional potency (DEGs), functional breadth (GSEA), and E3 dependency (WT/KO shift)—to define distinct compound archetypes. Specifically, we ultimately divided the compounds in the library into four tiers (Figure 5B). The first tier, representing potent molecular glues, consists of candidates with high potency in WT cells and a near-total loss of activity in KO cells, marking them as the highest priority for optimization. The second tier integrates intermediate and latent leads that exhibit moderate transcriptional activity or coordinated functional signals, representing promising seed scaffolds that may require further structural refinement to enhance potency. The third tier comprises active but E3-independent perturbators; these molecules induce significant transcriptional remodeling that remains largely unchanged in the KO background, suggesting they function via E3-independent mechanisms, such as traditional protein inhibition or kinase-like activities. Finally, the fourth tier includes inert or out-of-scope compounds that failed to meet significance thresholds across the evaluated parameters. A comprehensive overview of the tier assignments for all screened compounds is provided in Table S1. It should be noted that the threshold value and the definition criteria can be customized according to the specific requirements of the screening of different molecular glue compound libraries.

Overall, we envision that this transcriptomic profiling screening platform (Glue Perturbation Screen [GPS]) can be incorporated into the molecular glue discovery project at an early stage in order to assist in prioritizing new potential target degraders.

### Proteomic characterization and cross-validation confirm the high-fidelity predictive power of the GPS platform for candidate molecular glues

To further elucidate and validate the target selectivity of our prioritized candidates at the proteomic level, we selected LC-02-047-P1, a representative from the first-tier compound, and LC-02-051, a second-tier compound, for tandem mass tag (TMT)-based quantitative proteomics analysis in Hep3B cells. It’s illustrated in the volcano plot that LC-02-047-P1 induced a profound remodeling of the proteome, characterized by the marked downregulation of CSNK1A1 (CK1α), WIZ, and FAM83G (Figure 6A). From a mechanistic standpoint, CK1α emerges as the primary neosubstrate directly engaged by LC-02-047-P1. To validate these findings, immunoblotting analysis was performed across a panel of hepatocellular carcinoma cell lines, including Hep3B, Huh7, and HepG2. The results confirmed that LC-02-047-P1 elicits near-complete degradation of CK1α without affecting GSPT1 protein levels (Figures 6C, 6D, and S2). To rigorously scrutinize whether the downstream cellular effects of LC-02-047-P1 depend on GSPT1 degradation, we established a Hep3B hepatoma cell line stably expressing the non-degradable GSPT1 mutant (G575N). While the GSPT1 degradation effect of CC-90009 was completely abolished in G575N-expressing cells, the CK1α degradation effect of LC-02-047-P1 remained completely unperturbed (Figure S3). This provides definitive, protein-level evidence that LC-02-047-P1-induced cellular effects are entirely independent of GSPT1 degradation. Although WIZ exhibited significant depletion in the TMT proteomic data, its protein levels remained largely unaltered in the subsequent immunoblotting analysis.

**Figure 6:**
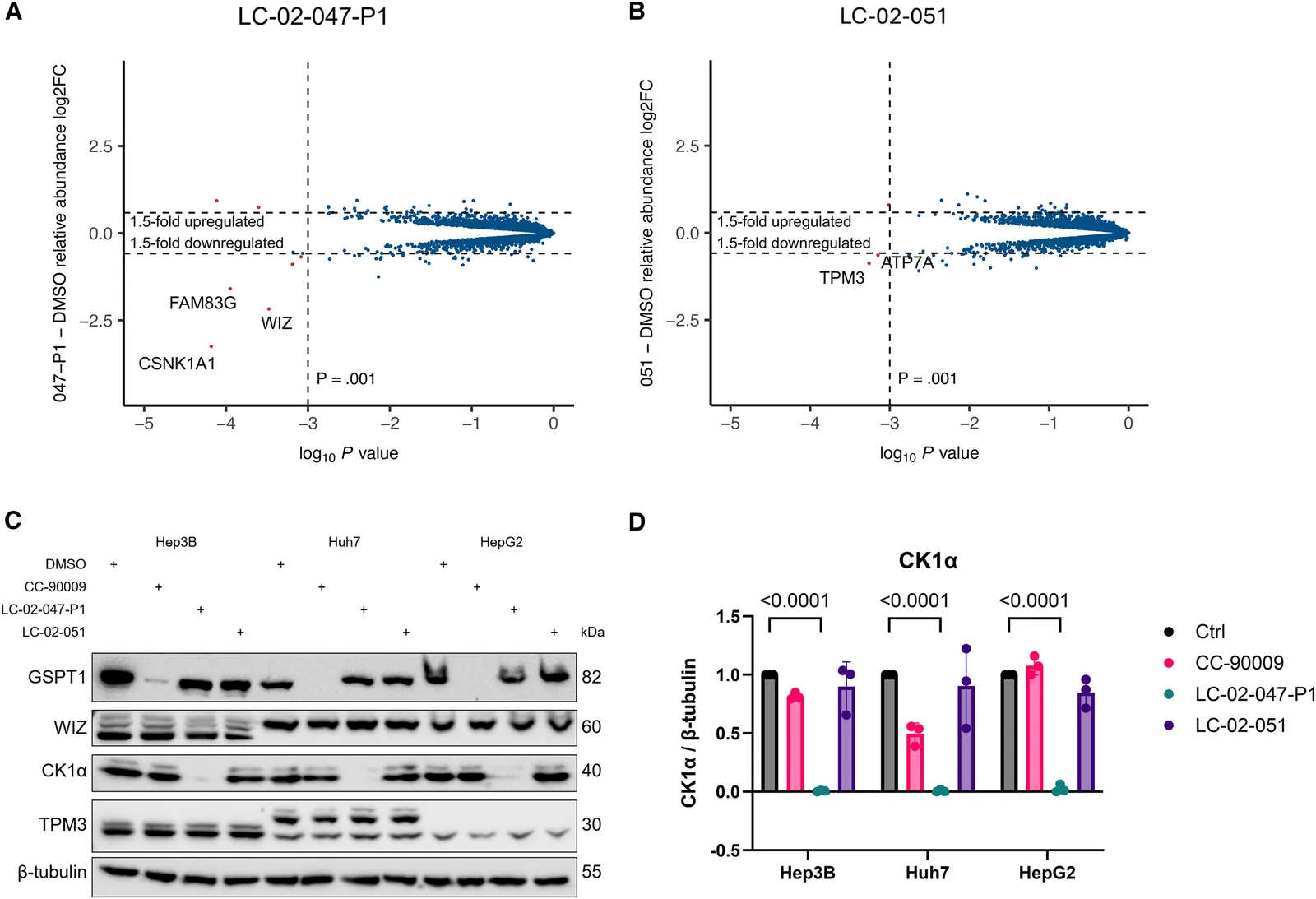
Proteomic profiling and immunoblot validation reveal target selectivity and neo-substrate degradation signatures of candidate molecular glues (A) Volcano plot showing TMT-based quantitative proteomics analysis in Hep3B cells following treatment with LC-02-047-P1 compared with DMSO control, highlighting the prominent downregulation of CSNK1A1 (CK1α). The LC-02-047-P1 and DMSO groups comprised two and three technical replicates, respectively. The horizontal dashed lines demarcate the 1.5-fold upregulation and downregulation thresholds, and the vertical dashed line indicates a significance cutoff of *p* = 0.001. (B) Volcano plot of TMT-based quantitative proteomics analysis in Hep3B cells treated with LC-02-051 relative to DMSO control, demonstrating a weaker proteomic perturbation profile with minor downregulation restricted to TPM3 and ATP7A. The LC-02-051 and DMSO groups comprised two and three technical replicates, respectively. (C) Representative western blot analysis of three independent biological experiments performed across a panel of hepatocellular carcinoma cell lines (Hep3B, Huh7, and HepG2) treated with DMSO, CC-90009, LC-02-047-P1, or LC-02-051 at a concentration of 1 μM for 18 h. Endogenous protein levels of GSPT1, WIZ, CK1α, and TPM3 were assessed, with β-tubulin serving as the loading control. Molecular weights (kDa) are indicated on the right. (D) Quantification of CK1α protein levels normalized to β-tubulin across Hep3B, Huh7, and HepG2 lines, confirming significant and selective near-complete degradation of CK1α mediated exclusively by LC-02-047-P1. Data are presented as mean ± SD (*n* = 3 independent biological replicates). Significant *p* values are indicated above the brackets, evaluated by two-way ANOVA. See also Figures S2–S4.

Conversely, the second-tier candidate LC-02-051 elicited a substantially weaker proteomic perturbation, with only TPM3 and ATP7A exhibiting minor downregulation (Figure 6B). Subsequent western blot validation of TPM3 revealed no statistically significant degradation across the tested cell lines (Figures 6C and S2), a finding perfectly aligned with its classification as a weaker, latent scaffold with modest transcriptional activity. Notably, the TMT proteomic data showed a general correspondence with the distribution and intensity of GPS signals, with compounds exhibiting stronger GPS signatures tending to display more pronounced neosubstrate degradation profiles (Figure S4). By demonstrating that GPS signals are closely associated with underlying degradation activities, these findings underscore the biological relevance of the GPS platform and strongly substantiate its utility as a robust, high-fidelity pipeline for early-stage efficacy prediction, tier stratification, and target deconvolution of novel molecular glue libraries.

## Discussion

The systematic discovery of MGDs has long been hindered by the lack of high-throughput methods that can simultaneously evaluate potency and E3 dependency. In this study, we addressed this gap by establishing an integrated transcriptomic screening framework. By leveraging the sensitivity of DRUG-seq2 and the rigor of isogenic CRBN WT/KO models, we successfully stratified a 117-compound library into distinct functional tiers, identifying bona fide CRBN-dependent glues while unmasking secondary off-target effects.

It is important to note that the commercial molecular glue can serve as a positive control. However, it does not imply the sharing of new substrate targets across the entire library. Instead, it establishes a reference archive derived from validated molecular glues. Given that their capabilities to induce E3 ligase-dependent protein degradation are well-characterized and their transcriptomic responses represent authentic molecular glue action modes. Consequently, while the 106 in-house synthesized compounds possess uncharacterized and potentially diverse targeting profiles, their bioactivity and phenotypic efficacy can be systematically evaluated and contextualized against the high-confidence boundaries defined by these positive reference standards.

In establishing the GPS platform, the selection of a 24-h incubation window for DRUG-seq2 profiling was a deliberate strategy optimized to maximize screening robustness and reproducibility. Although molecular glue-induced neosubstrate degradation typically initiates within a much shorter time frame, early-stage transcriptional changes are often modest in amplitude and highly susceptible to technical noise or experimental variability inherent to high-throughput screening environments. In contrast, the 24-h time point consistently yields stronger, more stable transcriptional signatures, which are essential for reliable compound classification and prioritization across large-scale libraries. It should be noted that the profiles captured at this endpoint represent an integrated cellular response—reflecting both the primary consequences of target degradation and downstream secondary transcriptional cascades—rather than an exclusive snapshot of immediate, primary degradation events. Consequently, the GPS platform is fundamentally designed to identify compounds that effectively drive holistic, molecular glue-associated cellular states.

The strategy for baseline selection is a pivotal factor in high-throughput transcriptomic screening, as the reference state directly dictates the signal-to-noise ratio for hit identification. We believe the strategy for defining an experimental baseline is inherently contingent upon the scale and format of the screen. A previous large-scale DRUG-seq study utilized a redundant small interfering RNA (siRNA) activity (RSA) statistic, where random selection of control wells was used to identify the subset that could minimize internal variations to the greatest extent.^15^ In such large-scale transcriptomic platforms where dozens of control wells are available, iterative sampling serves as a powerful statistical tool to identify the representative average of a given batch. For the 96-well DRUG-seq2 format, we adopted a conceptually similar but more streamlined approach by prioritizing intra-plate internal consistency through correlation-based filtering. While the iterative method thrives on large sample sizes to reach statistical convergence, our approach provides a direct measure of technical reproducibility when the number of replicates is constrained. Both strategies strive toward the same objective: identifying a high-fidelity reference state that reflects the unstimulated cellular transcriptome.

Also, our study highlights the importance of the cellular proteomic landscape in MGD discovery. The efficacy of a molecular glue is fundamentally dictated by its ability to induce a productive ternary complex between an E3 ligase and a neo-substrate.^4^^,^^28^^,^^29^ Given that the human genome encodes over 600 E3 ubiquitin ligases, each with distinct tissue- and cell-type-specific expression profiles, the selection of an appropriate screening model is paramount to avoiding false-negative results.^30^ The robust transcriptional signatures observed for CC-90009 in Hep3B cells are inherently linked to the endogenous abundance of both CRBN and respective targets. Therefore, our platform’s utility extends beyond a single cell line and provides a template for selecting abundance-matched models tailored to specific E3-substrate pairs, such as utilizing VHL-high or DCAF15-high genetic backgrounds for targeted screening.

To demonstrate the high-throughput viability of the GPS platform, we assessed its scalability when exceeding 1,000 compounds. The empirical cost was reduced to less than 100 RMB (∼$14 USD) per sample, amounting to merely ∼2% of that of standard TMT-proteomics. The end-to-end turnaround time remains highly efficient, requiring 4 days for upstream preparation, 14 days for library construction and sequencing, and 1 to 2 days for automated downstream bioinformatic characterization of potential molecular glue action signatures. Ultimately, this total 3-to-4-week workflow provides a highly accessible, cost-effective, and scalable alternative for large-scale chemical screening.

A central strength of our platform lies in the use of transcriptomic signature as a high-resolution proxy for cellular state. Compared to traditional proteomics, which is often limited by high costs and lower throughput, transcriptomic profiling provides a broader view of the functional consequences of protein degradation. Our results demonstrate that potent compounds can clearly cause significant transcriptional perturbations. Even in the absence of robust differential gene expression, GSEA-based pathway enrichment can capture coordinated biological shifts, identifying potential bioactivity in molecules that would otherwise be discarded as inactive. This sensitivity is crucial for the early-stage optimization of molecular glue seeds, where nascent biological signals may be subtle but mechanistically significant.

We therefore propose that tier-2 compounds constitute potential molecular glue seeds that may operate under several distinct mechanistic scenarios, including (1) compounds with weaker E3 ligase engagement or suboptimal ternary complex formation that generate only a partial molecular glue-associated transcriptional signature; (2) compounds acting through novel degradation mechanisms or neosubstrates that are underrepresented in the current training set; and (3) compounds that induce weaker or more context-dependent transcriptional responses in the tested cell lines, thereby limiting the strength of their GPS signatures despite potential biological activity.

However, we acknowledge that transcriptomic signatures remain an indirect measure of protein-level events. While we have demonstrated that transcriptional signatures can accurately reflect E3 dependency, candidate leads identified through this platform should ultimately be validated via quantitative proteomics and structural biology to confirm direct ternary complex formation. Nevertheless, the scalability and precision of our tiered classification system offer a robust solution for the rapid prioritization of synthetic libraries. This strategy effectively bridges the gap between high-volume chemical screening and low-throughput mechanistic validation, providing a versatile roadmap for the rational discovery of the next generation of targeted protein degraders.

### Limitations of the study

A limitation of the current screening framework is our reliance on a single 24-h window for transcriptomic analysis. While this duration is highly effective for ensuring signal reproducibility and robust library stratification, it captures an integrated cellular state that combines direct target-degradation signatures with secondary downstream events and cellular stress cascades. This can potentially mask the primary, immediate transcriptional footprints driven directly by neosubstrate depletion. Future iterations of this work will benefit from incorporating temporal or kinetic transcriptional profiling across multiple, earlier time points. Implementing such time-resolved analysis will be instrumental in chronologically dissecting early degradation-driven events from later secondary adaptive, compensatory, or stress responses, thereby enhancing the overall mechanistic depth of candidate characterization.

## Resource availability

### Lead contact

• Requests for further information and resources should be directed to and will be fulfilled by the lead contact, Wenchao Wu (wuwch@sysucc.org.cn).

### Materials availability

• This study did not generate new unique reagents.
• All compounds used in this study are available from the lead contact upon reasonable request.

### Data and code availability

• DRUG-seq2 data have been deposited at NCBI Sequence Read Archive (SRA) at PRJNA1437501 and are publicly available as of the date of publication.
• Proteomics data have been deposited at iProX at IPX0017864000 and are publicly available as of the date of publication.
• This paper does not report original code.
• Any additional information required to reanalyze the data reported in this paper is available from the lead contact upon request.

## Acknowledgments

The authors thank David Weinstock for thoughtful comments. They also thank X.D. for valuable input and advice on the bioinformatics analyses. The authors are grateful to the staff of Plastech Pharmaceutical Technology Co., Ltd., for technical support with Drug-seq2 library preparation, and to the staff of Jingjie PTM-Biolab Co., Ltd., for performing the TMT-proteomics. They thank B.J. and W.L. for providing the compound library. This work was supported in part by the National Program for Introducing Talents from abroad (grant no. KY022467 to W.W.), and the 10.13039/501100001809National Natural Science Foundation of China (grant no. 82574250 to B.J.).

## Author contributions

W.W. and B.J. conceived and designed the study. L.C., W.L., and B.J. designed and synthesized the compounds. D.H., Z.S., H.O., L.H., and P.Y. performed the experiments. D.H. and M.S. analyzed and interpreted the data. D.H. and X.D. conducted the bioinformatic analyses. D.H. and W.W. drafted the manuscript. All authors critically revised the manuscript and approved the final version.

## Declaration of interests

The authors declare no competing interests.

## STAR★Methods

### Key resources table

**Table:** 

REAGENT or RESOURCE	SOURCE	IDENTIFIER
**Antibodies**		

Cas9	Cell Signaling Technology	Cat#19526T
GSPT1	Proteintech	RRID:AB_2115506
CRBN	Novus	RRID:AB_11037820
GAPDH	Cell Signaling Technology	RRID:AB_10622025
CSNK1A1	Proteintech	RRID:AB_11183034
TPM3	Proteintech	RRID:AB_2205671
WIZ	Invitrogen	RRID:AB_11154149
β-tubulin	Cell Signaling Technology	RRID:AB_823664
Goat Anti-Rabbit IgG-HRP Conjug	BIO-RAD	RRID:AB_11125142
Goat Anti-Mouse IgG-HRP Conjug	Invitrogen	RRID:AB_2533947

**Bacterial and virus strains**		

StbI3	Daling	Cat#DLC106

**Chemicals, peptides, and recombinant proteins**		

Eragidomide(CC-90009)	MCE	Cat#HY-130800; CAS:
SJ6986	MCE	Cat#HY-132199; CAS: 2765625-93-0
TMX-4116	MCE	Cat#HY-145322; CAS: 2766385-56-0
BMS-986463	MCE	Cat#HY-175749; CAS: 3025467-07-3
MYC degrader 1	MCE	Cat#HY-162318; CAS: 2946670-96-6
Lenalidomide	MCE	Cat#HY-A0003; CAS: 191732-72-6
DEG-77	MCE	Cat#HY-157334; CAS: 3032265-06-5
ZXH-1-161	MCE	Cat#HY-147158; CAS: 2407654-51-5
SJ3149	MCE	Cat#HY-160444; CAS: 3026986-17-1
BMS-986397	MCE	Cat#HY-159646; CAS: 2564486-44-6
MRT-2359	MCE	Cat#HY-153356; CAS: 2803881-11-8
CC-92480	MCE	Cat#HY-129395; CAS: 2259648-80-9
DMSO	Sigma-Aldrich	Cat#D2650
HEPES	Sigma-Aldrich	Cat#H3375
PEI	MCE	Cat#HY-K2014

**Critical commercial assays**		

BCA Protein Assay Kit	Thermo Fisher Scientific	Cat#23227
AQueous One Solution Cell Proliferation Assay	Lifeman	Cat#G3580

**Deposited data**		

DRUG-seq2 raw data	This paper	SRA: PRJNA1437501,
Proteomics raw data	This paper	iProX: IPX0017864000

**Experimental models: Cell lines**		

Hep3B	Jennio Biotech	RRID:CVCL_0326
Huh7	Jennio Biotech	RRID:CVCL_0336
HepG2	Jennio Biotech	RRID:CVCL_0027
293T	ATCC	RRID:CVCL_0063

**Oligonucleotides**		

CRBN sgRNA: 5′-CGGGCGGTTACCAGGCAGGAG-3′	This paper	N/A

**Software and algorithms**		

edgeR	Chen et al.^31^	RRID:SCR_012802
DESeq2	Love et al.^32^	RRID:SCR_015687
clusterProfiler	Wu et al.^33^	RRID:SCR_016884
GraphPad Prism 10	Dotmatics	RRID:SCR_002798
Image Lab	Bio-Rad	RRID:SCR_014210

### Experimental model and study participant details

Hep3B, Huh7 and HepG2 hepatocellular carcinoma cell lines (GuangZhou Jennio Biotech Co., Ltd., Guangzhou, China) were cultured in Dulbecco’s modified Eagle’s medium (DMEM; Cat#11054001, Gibco) supplemented with 10% fetal bovine serum (FBS; Cat#F8318, SigmaAldrich) and 1% penicillin/streptomycin (Cat#15140122, Gibco) at 37°C under 5% CO_2_. The 293T packaging cell line (ATCC, CRL-3216) was used for lentiviral production. Cell lines were authenticated by short tandem repeat (STR) profiling and tested negative for mycoplasma contamination prior to use.

### Method details

#### Lentivirus production

Lentiviral particles were produced in 293T cells grown in 90-mm dishes to ∼70% confluence. Transfections were performed using 64 μL of PEI (MCE, Cat#HY-K2014) with a plasmid mixture consisting of 2 μg of VSV-G, 6 μg of PAX2, 8 μg of transgene. Culture medium was refreshed 24 hours post-transfection and viral supernatant collected 48 hours later, filtered through a 0.45 μm filter, aliquoted and stored at −80°C.

#### Generation of CRBN wild-type (WT) and knockout (KO) cell lines

Hep3B cells were transduced with pLv5-Cas9-Neo to establish stable Cas9-expressing lines (Hep3B + Cas9) and were selected with G418 (500 ng/mL) for 10 days. To generate *CRBN* knockouts, target-specific single-guide RNAs (sgRNAs) were cloned into the doxycycline-inducible FgH1tUTG vector (Addgene, Cat#70183), which co-expresses EGFP for selection. Hep3B + Cas9 cells were transduced with the FgH1tUTG-sgRNA constructs; sgRNA expression was induced by doxycycline (2 μg/mL) for 3 days, and EGFP-positive cells were sorted by fluorescence-activated cell sorting (FACS; BD FACSAria II) to obtain sgRNA-expressing single clones.

The sgRNA sequences used were: *CRBN*, 5’-CGGGCGGTTACCAGGCAGGAG-3’.

#### Generation of GSPT1 G575N mutant Hep3B cell line

The open reading frame (ORF) of wild-type GSPT1 was cloned into the pLVX-EF1A-puro vector. The G575N point mutation was then introduced using the NEB HiFi DNA Assembly Kit (NEB, #E2621S) according to the manufacturer’s instructions. The GSPT1-G575N mutant construct was verified by Sanger sequencing. To generate lentiviral particles, HEK293T cells cultured in 100-mm dishes were grown to approximately 70% confluence and transfected with a mixture of 18 μL FuGENE HD (Promega, #E2311), 2 μg pMD2.G (VSV-G), 2 μg psPAX2, and 2 μg of either pLVX-EF1A-GSPT1-WT or pLVX-EF1A-GSPT1-G575N plasmid. Viral supernatants were harvested 48 hours post-transfection. Hep3B cells were subsequently transduced with the viral supernatants in the presence of polybrene to enhance transduction efficiency, followed by selection with 2 μg/mL puromycin. Expression of wild-type and G575N mutant GSPT1 in Hep3B cells was confirmed by Western.

#### Western Blot

Total protein was extracted from cells using Cell Extraction buffer (ThermoFisher, FNN0011) supplemented with protease and phosphatase inhibitor cocktail (ThermoFisher, 78442). Protein concentrations were determined using a BCA Protein Assay Kit (ThermoFisher, 23227). Equal amounts of protein (20–30 μg per lane) were separated by SDS-PAGE (Beyotime) and subsequently transferred onto NC membranes (Merck Millipore, HATF00010).After blocking with 5% skim milk (BD, 232100) at room temperature for 1 h, the membranes were incubated overnight at 4°C with primary antibodies against CRBN (1:3000, Novus, NBP1-91810), GSPT1 (1:3000, Proteintech, 10763-1-AP)and GAPDH (1:5000, CST, 5174S) as a loading control. The following day, membranes were incubated with HRP-conjugated secondary antibodies for 1 h. Protein bands were visualized using enhanced chemiluminescence (ECL) reagents (Cytiva, RPN2232) and captured with a chemi-luminescence imaging system (Bio-red).

#### Cell viability and IC50 determination

For dose-response studies and IC50 determination, cells were seeded in 96-well plates (triplicate wells per condition). Eight hours after seeding, serial dilutions of CC-90009 were added. Cell viability was measured 72 hours after treatment using the AQueous One Solution Cell Proliferation Assay according to the manufacturer’s instructions; absorbance was read on a microplate reader. IC50 values were calculated from nonlinear regression fits of dose-response curves.

#### Compound treatment

Hep3B cells were seeded in 96-well plates at a density of 5000 to 10000 cells per well and allowed to adhere for at least 24 h. Subsequently, cells were treated with 1 μM of the compound or an equivalent volume of DMSO (vehicle control). All treatments were performed in triplicate. After 24 h, 96-well plates were collected and cells were lysed for DRUG-seq2 library preparation.

#### DRUG-seq library construction and sequencing

DRUG-seq2 data were processed to obtain expression matrix of gene counts according to drop-seq analysis protocol (https://github.com/broadinstitute/Drop-seq). Briefly, reads for each well were split through 10-base well and 10-base plate barcodes and then mapped to the human transcriptome with the GRCh38 reference index and Ensembl gene references by STAR.39 The unique alignments were further demultiplexed based on the transcript UMI for each gene. The UMIs of each gene were counted as its raw counts. CPM (counts of exon model per million mapped reads) matrix was transferred from the raw count matrix and used for hierarchical clustering, cluster heatmap, and PCA analysis, Our method for normalization with VST (default): first, use loess to fit a straight line to log (variance) and log (mean), then use the observed mean and expected variance to normalize the gene expression, and finally calculate the variance according to the normalized expression with the largest reservation. Drug-seq2 experiments were conducted by Plastech Pharmaceutical Technology Co. LTD by using Elite CellSignature High-Throughput 3' Transcriptome kit (Plastech, XR2302), with a reported protocol.^16^

#### Proteomic analysis

The sample was grinded with liquid nitrogen and lysed using lysis buffer (8 M urea, 1% protease inhibitor cocktail), followed by sonication on ice and centrifugation at 12,000 g at 4 °C for 10 min; the supernatant was collected and protein concentration determined with a BCA kit. Proteins were precipitated with 20% (m/v) TCA, washed with pre-cooled acetone, and redissolved in 200 mM TEAB. Trypsin was added at a 1:50 trypsin-to-protein mass ratio for overnight digestion. The sample was reduced with 5 mM dithiothreitol (30 min at 56 °C), alkylated with 11 mM iodoacetamide (15 min at room temperature in darkness), and desalted by a Strata X SPE column. Tryptic peptides were labeled with TMT reagents (ThermoFisher Scientific) for 2 hours at room temperature, quenched by adding 5% hydroxylamine, and fractionated by high pH reverse-phase HPLC using an Agilent 300 Extend C18 column into 12 combined fractions. LC-MS/MS analysis was performed on an EASY-nLC 1200 UPLC system coupled to an Orbitrap Fusion Lumos mass spectrometer. Peptides were loaded onto a home-made reversed-phase analytical column (25-cm length, 100 μm i.d.) and separated with a 120-min gradient of solvent A (0.1% FA, 2% ACN) and solvent B (0.1% FA, 90% ACN) at 600 nL/min. In DDA mode, full MS scan resolution was set to 120,000 (scan range of 400–1600 m/z), and the top 20 precursor ions were fragmented via CID (collision energy of 35%) and secondary HCD (collision energy of 55%) with an MS/MS resolution of 50,000. MS/MS data were processed using Proteome Discoverer (v.2.4) against the Homo_sapiens database with trypsin allowing up to 2 missing cleavages. Carbamidomethyl on Cys was specified as fixed modification, while oxidation on Met and acetylation on protein N-terminal were variable modifications (FDR < 1%).

#### Bioinformatics

DRUG-seq data processing was carried out with a custom script. Raw sequencing counts were analyzed using R software (version 4.4.3). To remove noise from lowly expressed genes, the filterByExpr function from the edgeR package^31^ was applied, which automatically determines a threshold based on library size and experimental design. A sample-to-sample distance heatmap was generated using pheatmap package. The filtered data were then normalized, and differential expression analysis was performed using the DESeq2 package.^32^ Genes with an absolute log2 fold change (|log2FC|) > 1 and an adjusted *P*-value (padj) < 0.05 were identified as differentially expressed genes (DEGs). Gene Set Enrichment Analysis (GSEA) were performed using the clusterProfiler^33^^,^^34^ and msigdbr packages. Gene symbol mapping was facilitated by the org.Hs.eg.db database. Data visualization, including volcano plots and complex heatmaps, was implemented using ggplot2, ggrepel, and ComplexHeatmap.

### Quantification and statistical analysis

Quantitative data are presented as mean ± standard deviation (SD) unless otherwise indicated in the figure legends. Data presented in Figure 2G are shown as mean ± standard error of the mean (SEM). Data were tested using a 2-tailed Student’s t test (when comparing 2 groups). For multiple group comparisons, one-way or two-way analysis of variance (ANOVA) was performed, followed by Tukey’s or Dunnett’s post hoc test for multiple comparisons, as specified in the figure legends. For all tests, significance was determined with a 95% confidence interval on GraphPad Prism, version 10.
