# Supplementary material for: A scalable, high-throughput glue perturbation screening platform for molecular glue discovery
Source: iScience. 2026 Sep 19;29(10):117600. doi: 10.1016/j.isci.2026.117600 (PMC13628600; doi:10.1016/j.isci.2026.117600)
Supplement: Supplementary file 1 [file mmc1.pdf]

iScience, Volume *29*

Supplemental information

A scalable, high-throughput glue perturbation screening platform for molecular glue discovery

Diyun Huang, Zeyu Shuang, Lu Chen, Hanyue Ouyang, Peiyu Yang, Lichang Huang, Wenchao Lu, Ming Shi, Xiaofan Ding, Baishan Jiang, and Wenchao Wu

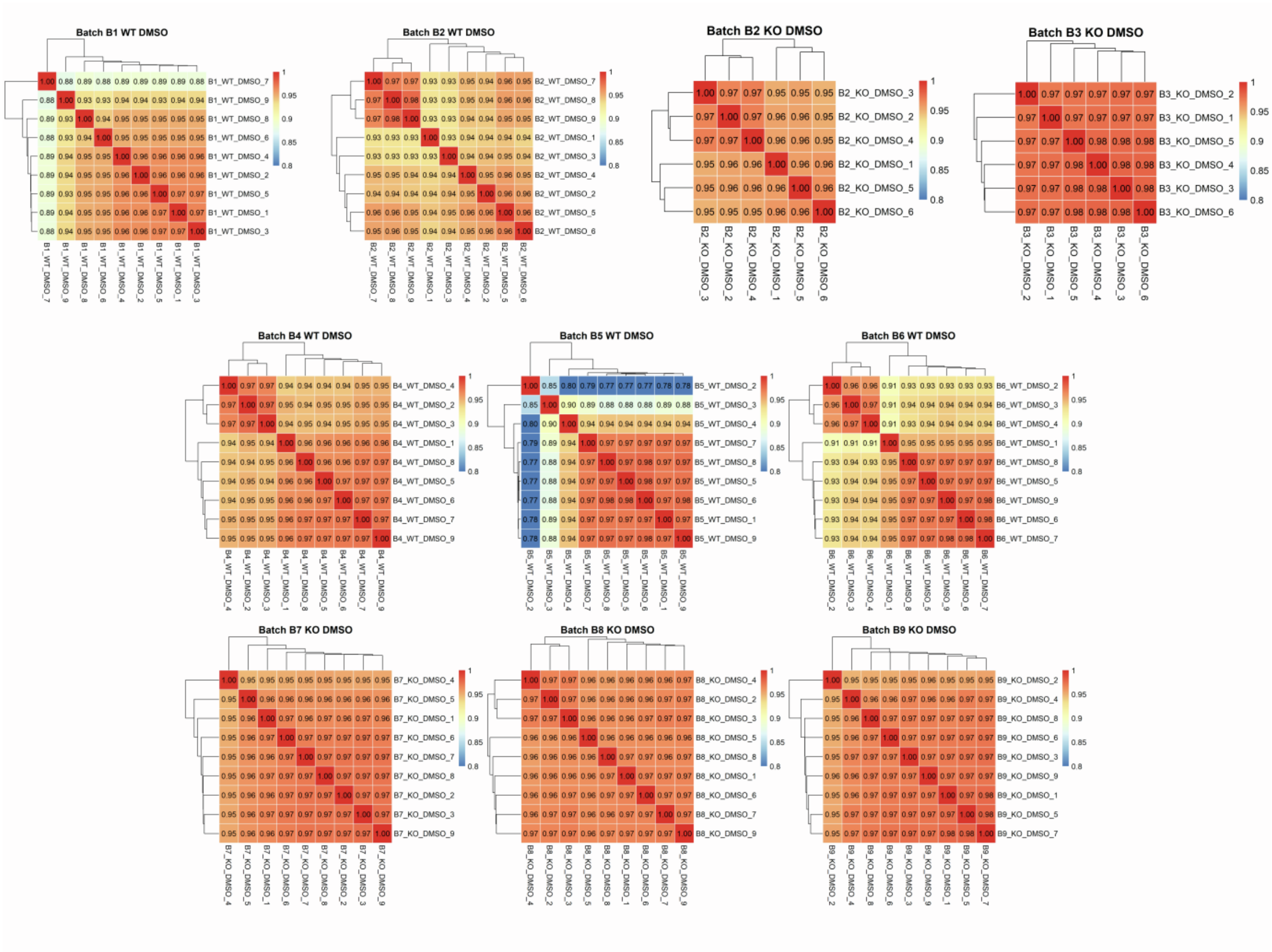

Figure S1 | Quality control and baseline establishment for DRUG-seq2 profiling.

Pairwise Pearson correlation heatmaps of DMSO control replicates across multiple experimental batches. Each cell in the heatmap represents the correlation coefficient between the transcriptomic profiles of two DMSO replicates within the same 96-well plate. Replicates exhibiting a correlation coefficient of $r \geq 0.95$ were defined as high-consensus wells.

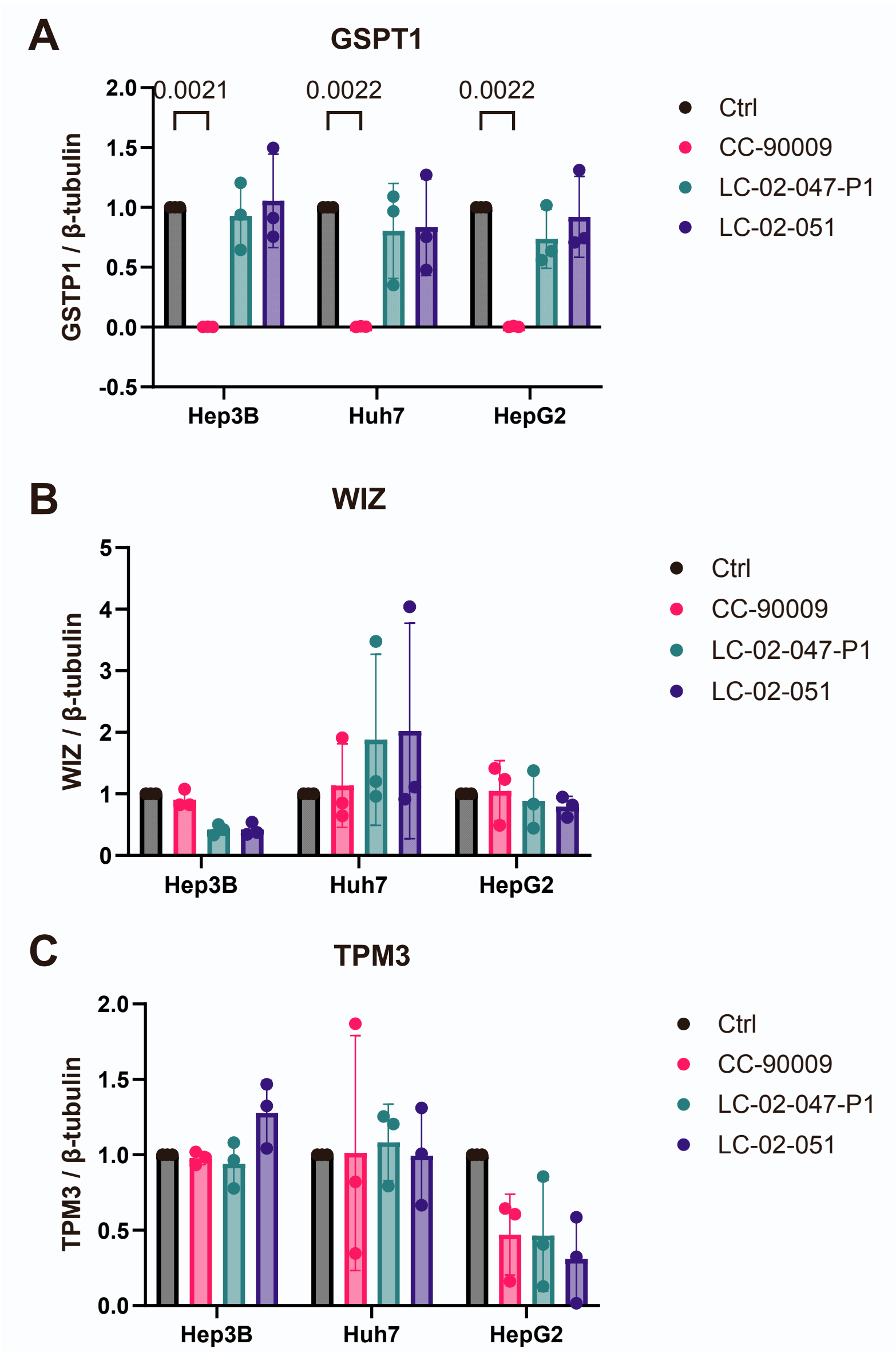

Figure S2 | Statistical Quantification of GSPT1, WIZ, and TPM3 Protein Abundance Across Hepatocellular Carcinoma Cell Lines.

Hep3B, Huh7, and HepG2 cells were treated with DMSO, CC-90009, LC-02-047-P1 or LC-02-051 (1 μM) for 18 h prior to cell lysis.

(A) Quantification of GSPT1 protein levels normalized to β-tubulin in Hep3B, Huh7, and HepG2 cells treated with DMSO (Ctrl), CC-90009, LC-02-047-P1, or LC-02-051, validating selective and profound GSPT1 degradation restricted to the positive control CC-90009.

(B) Relative protein abundance quantification of WIZ normalized to β-tubulin under the same treatment conditions across the three cell lines.

(C) Relative protein abundance quantification of TPM3 normalized to β-tubulin across Hep3B, Huh7, and HepG2 lines.

Data are presented as mean ± SD (n = 3 independent biological replicates). Significant *p*-values are indicated above the brackets, evaluated by two-way ANOVA.

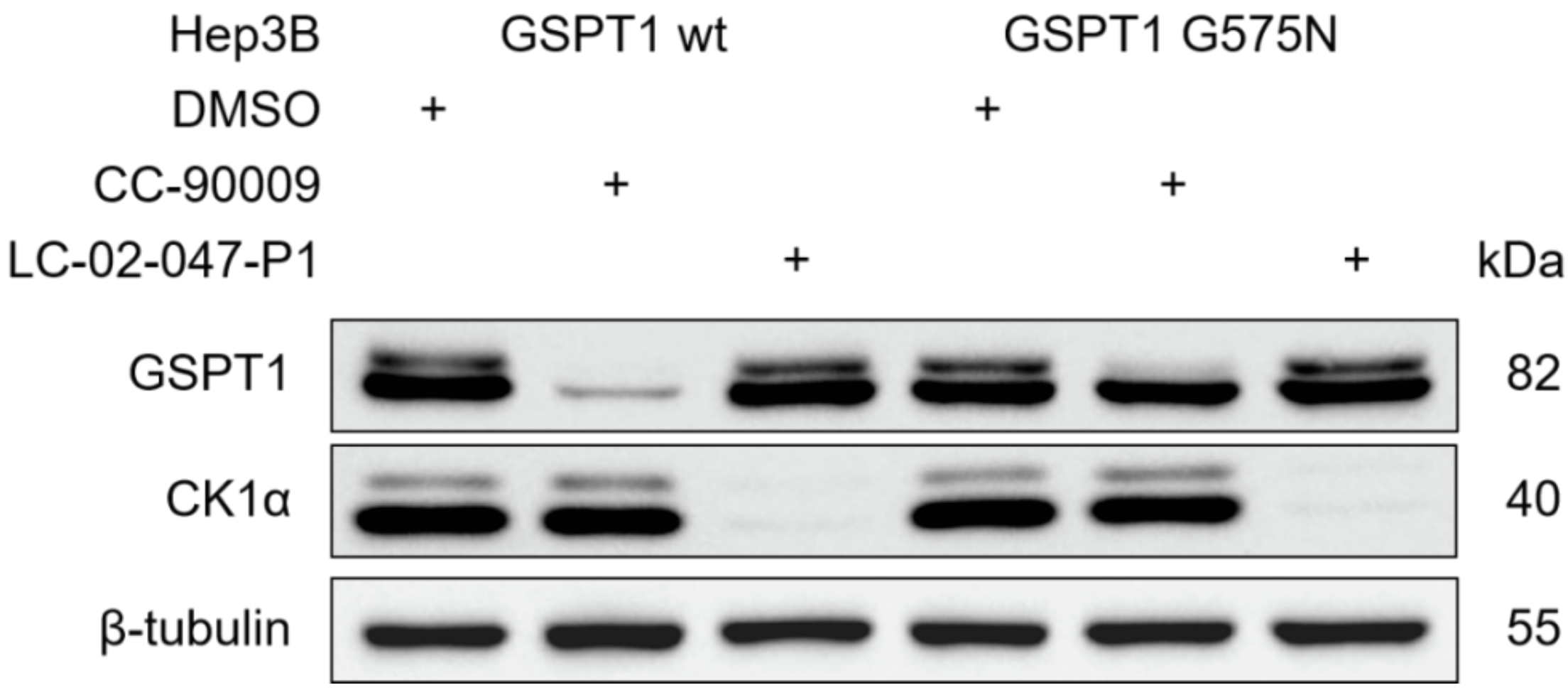

Figure S3 | LC-02-047-P1 - induced CK1α degradation operates independently of GSPT1 proteolysis. Western blot analysis of GSPT1 and CK1α protein levels in Hep3B cells stably overexpressing either wild-type GSPT1 (GSPT1 wt) or the non-degradable GSPT1 mutant (GSPT1 G575N). Cells were treated with DMSO, CC-90009, or LC-02-047-P1 1 μM for 18 h prior to cell lysis. β-tubulin was utilized as the loading control, and molecular weights (kDa) are indicated on the right. Representative western blot data were from three independent biological experiments.

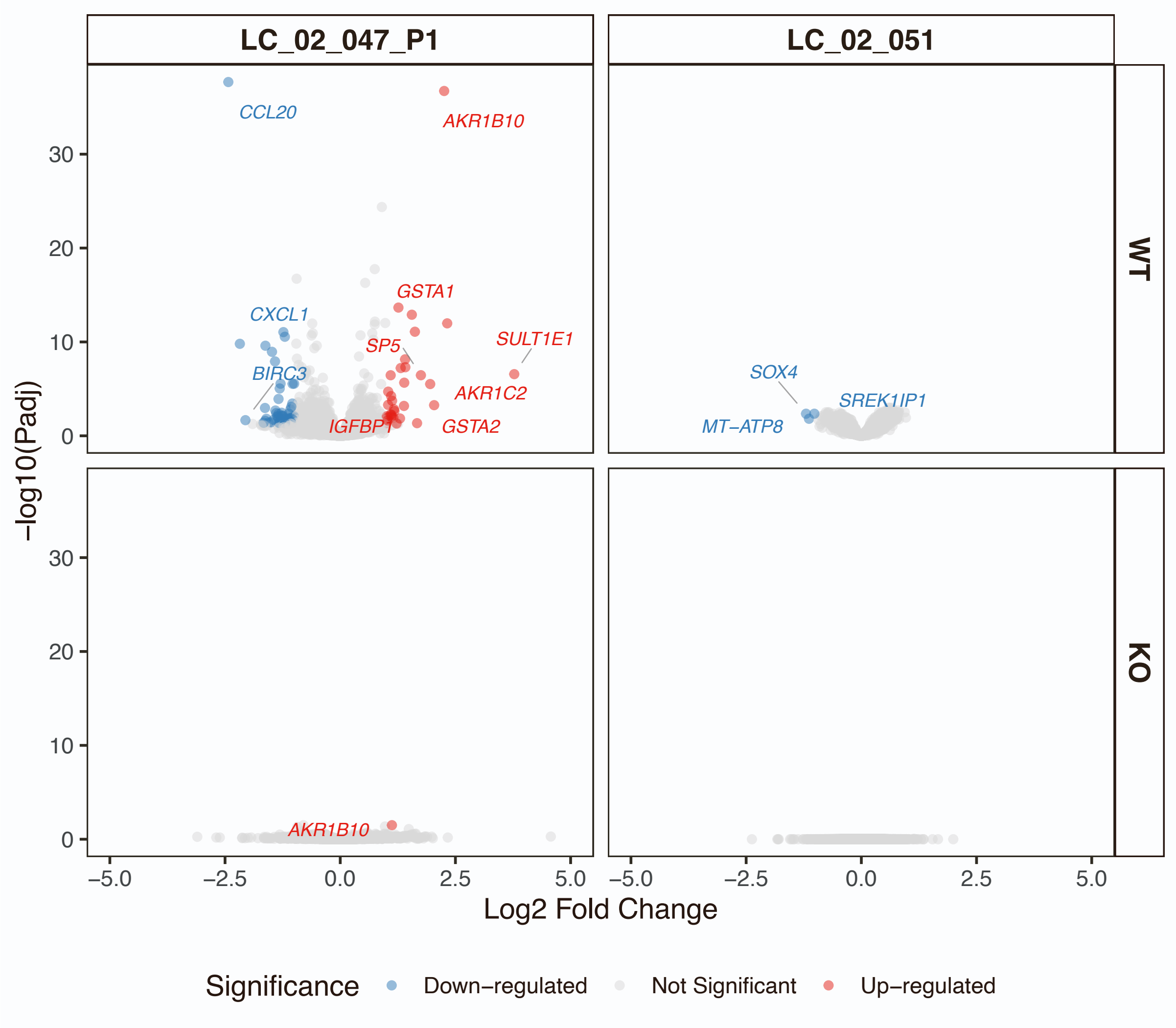

Figure S4 | Volcano plots comparing LC-02-047-P1 and LC-02-051 across WT and CRBN-KO cells. Hep3B cells were treated with DMSO, LC-02-047-P1 or LC-02-051 (1 μM) for 24h, followed by Drug-seq analysis. The volcano plot highlights the top 10 significantly regulated genes.

Table S1. Compound Tier Classification By GPS

name	DEG_in_WT	DEG_in_KO	pathway_in_WT	pathway_in_KO	Tier
SJ3149	4411	0	240	0	Tier 1
Lenalidomide	2025	1	121	0	Tier 1
BMS_986397	451	1	106	0	Tier 1
MYC degrader 1	354	2	10	3	Tier 1
CC_90009	171	0	84	38	Tier 1
LC_04_118	148	19	237	178	Tier 1
LC_02_047_P1	99	1	29	1	Tier 1
DEG_77	79	1	145	0	Tier 1
BMS_986463	62	0	194	21	Tier 1
LC_03_104	58	0	3	0	Tier 1
G1	47	1	0	0	Tier 2
LC_04_078	38	0	2	0	Tier 2
Z1	37	2	134	0	Tier 2
LC_04_077	34	3	9	9	Tier 2
MRT_2359	34	1	2	2	Tier 2
LC_03_109	31	0	5	1	Tier 2
LC_03_103	31	1	3	0	Tier 2
LC_03_099	29	1	15	0	Tier 2
LC_03_108	28	1	2	0	Tier 2
LC_03_100	27	1	2	0	Tier 2
LC_03_105	25	4	7	0	Tier 2
LC_02_121	24	15	53	10	Tier 2
LC_03_184	24	1	0	0	Tier 2
LC_04_081	22	0	9	0	Tier 2
LC_03_098	22	2	0	0	Tier 2
LC_03_200	22	10	0	0	Tier 2
LC_03_097	19	5	1	0	Tier 2
LC_03_107	17	0	4	0	Tier 2
LC_03_187	17	2	1	0	Tier 2
LC_03_055	13	1	5	0	Tier 2
LC_03_110	11	0	1	1	Tier 2
LC_04_114	10	0	2	0	Tier 2
LC_03_173	10	1	0	0	Tier 2
ZXH_1_16	10	1	0	0	Tier 2
LC_02_030	9	0	23	6	Tier 2
LC_03_160	9	1	1	0	Tier 2
K1	7	3	0	0	Tier 2
LC_03_078	7	2	0	0	Tier 2
LC_03_181	6	1	3	2	Tier 2

LC_03_194	5	0	10	0	Tier 2
LC_04_049	5	4	2	0	Tier 2
LC_03_197	5	0	0	0	Tier 2
LC_03_066	4	0	35	0	Tier 2
LC_02_036	4	0	7	0	Tier 2
LC_02_119	4	3	2	1	Tier 2
LC_03_157	4	0	2	0	Tier 2
LC_04_075	4	0	2	0	Tier 2
LC_02_051	3	0	136	0	Tier 2
LC_03_065	3	1	26	0	Tier 2
LC_03_064	3	0	5	1	Tier 2
LC_02_104	3	0	2	0	Tier 2
LC_02_150	3	1	0	0	Tier 2
LC_03_062	3	0	0	0	Tier 2
LC_03_112	2	0	25	8	Tier 2
TMX_4116	1	0	13	0	Tier 2
LC_03_060	1	0	0	0	Tier 2
LC_03_152	60	1	6	7	Tier 3
LC_03_102	58	3	3	13	Tier 3
LC_02_120	47	109	0	15	Tier 3
LC_05_004	26	51	40	17	Tier 3
LC_02_031	24	1	62	78	Tier 3
CC_92480	22	12	85	175	Tier 3
LC_04_048	20	4	1	9	Tier 3
LC_02_105	16	328	19	68	Tier 3
LC_04_087	16	4	6	37	Tier 3
LC_03_101	16	15	4	12	Tier 3
LC_04_047	13	3	1	16	Tier 3
LC_03_106	13	1	0	10	Tier 3
LC_04_040	10	0	0	3	Tier 3
LC_02_035	9	24	20	23	Tier 3
LC_04_028	6	21	23	30	Tier 3
LC_02_117	6	0	0	4	Tier 3
LC_02_148	5	10	22	306	Tier 3
LC_02_037	5	0	2	18	Tier 3
LC_02_118	5	42	1	41	Tier 3
LC_02_116	5	1	0	3	Tier 3
LC_04_041	5	1	0	31	Tier 3
LC_04_042	5	1	0	2	Tier 3
LC_02_033	4	106	5	32	Tier 3
W1	4	38	4	69	Tier 3

LC_03_111	4	0	1	10	Tier 3
LC_02_020	4	0	0	6	Tier 3
LC_03_177	4	0	0	2	Tier 3
LC_03_052	3	27	58	20	Tier 3
LC_03_067	2	0	0	8	Tier 3
SJ6986	2	11	0	1	Tier 3
LC_03_077	1	5	8	0	Tier 3
LC_03_068	1	12	0	16	Tier 3
LC_04_045	5	NA	22	NA	Tier 4
LC_02_158	4	NA	5	NA	Tier 4
LC_04_011	3	NA	28	NA	Tier 4
LC_03_063	3	3	0	0	Tier 4
LC_03_175	3	3	0	0	Tier 4
LC_04_014	1	NA	19	NA	Tier 4
LC_03_096	1	1	0	0	Tier 4
LC_03_035	0	0	51	0	Tier 4
LC_04_039	0	NA	19	NA	Tier 4
LC_04_008	0	NA	16	NA	Tier 4
LC_02_069	0	NA	12	NA	Tier 4
LC_03_034	0	NA	10	NA	Tier 4
LC_01_147	0	NA	5	NA	Tier 4
LC_02_032	0	NA	2	NA	Tier 4
LC_02_040	0	NA	1	NA	Tier 4
LC_02_135	0	0	1	0	Tier 4
LC_03_032	0	NA	1	NA	Tier 4
LC_03_036	0	NA	1	NA	Tier 4
LC_03_048	0	NA	1	NA	Tier 4
C1	0	1	0	20	Tier 4
LC_02_039	0	NA	0	NA	Tier 4
LC_03_033	0	NA	0	NA	Tier 4
LC_03_042	0	0	0	21	Tier 4
LC_03_047	0	NA	0	NA	Tier 4
LC_03_059	0	0	0	0	Tier 4
LC_03_061	0	0	0	0	Tier 4
LC_03_163	0	2	0	1	Tier 4
LC_04_017	0	NA	0	NA	Tier 4
LC_04_158	0	NA	0	NA	Tier 4
